# Baseline Repeated Measures from Controlled Human Exposure Studies: Associations between Ambient Air Pollution Exposure and the Systemic Inflammatory Biomarkers IL-6 and Fibrinogen

**DOI:** 10.1289/ehp.0900550

**Published:** 2009-09-29

**Authors:** Aaron M.S. Thompson, Antonella Zanobetti, Frances Silverman, Joel Schwartz, Brent Coull, Bruce Urch, Mary Speck, Jeffrey R. Brook, Michael Manno, Diane R. Gold

**Affiliations:** 1 Gage Occupational and Environmental Health Unit, University of Toronto, Toronto, Ontario, Canada;; 2 St. Michael’s Hospital, Toronto, Ontario, Canada;; 3 Department of Environmental Health, Harvard School of Public Health, Boston, Massachusetts, USA;; 4 Environment Canada, Toronto, Ontario, Canada

**Keywords:** air pollution, epidemiology, fibrinogen, inflammation, interleukin-6

## Abstract

**Introduction:**

Systemic inflammation may be one of the mechanisms mediating the association between ambient air pollution and cardiovascular morbidity and mortality. Interleukin-6 (IL-6) and fibrinogen are biomarkers of systemic inflammation that are independent risk factors for cardiovascular disease.

**Objective:**

We investigated the association between ambient air pollution and systemic inflammation using baseline measurements of IL-6 and fibrinogen from controlled human exposure studies.

**Methods:**

In this retrospective analysis we used repeated-measures data in 45 nonsmoking subjects. Hourly and daily moving averages were calculated for ozone, nitrogen dioxide, sulfur dioxide, and particulate matter ≤ 2.5 μm in aerodynamic diameter (PM_2.5_). Linear mixed-model regression determined the effects of the pollutants on systemic IL-6 and fibrinogen. Effect modification by season was considered.

**Results:**

We observed a positive association between IL-6 and O_3_ [0.31 SD per O_3_ interquartile range (IQR); 95% confidence interval (CI), 0.08–0.54] and between IL-6 and SO_2_ (0.25 SD per SO_2_ IQR; 95% CI, 0.06–0.43). We observed the strongest effects using 4-day moving averages. Responses to pollutants varied by season and tended to be higher in the summer, particularly for O_3_ and PM_2.5_. Fibrinogen was not associated with pollution.

**Conclusions:**

This study demonstrates a significant association between ambient pollutant levels and baseline levels of systemic IL-6. These findings have potential implications for controlled human exposure studies. Future research should consider whether ambient pollution exposure before chamber exposure modifies IL-6 response.

The association between air pollution and cardiovascular morbidity and mortality is supported by epidemiologic and experimental evidence (Bhatnagar 2006; Brook et al. 2004; Delfino et al. 2005; Dockery et al. 1993; Dominici et al. 2006; Donaldson et al. 2001; Petrovic et al. 2000; Samet et al. 2000; Schwartz 1999). Systemic inflammation may be one of the potential mechanisms mediating the effect of air pollution on cardiovascular outcomes (Pekkanen et al. 2000; Peters et al. 1997, 2001a, 2001b; Rückerl et al. 2007; Schwartz 2001; Seaton et al. 1999).

Elevated systemic levels of interleukin-6 (IL-6) have been demonstrated to be an independent risk factor for cardiovascular morbidity and mortality (Cesari et al. 2003; Volpato et al. 2001). IL-6 also stimulates the production of acute-phase proteins, including C-reactive protein (CRP), which can predict cardiovascular risk (Ridker et al. 2000). The literature documents pollutant-associated elevations in pulmonary IL-6 in healthy subjects (*in vivo*) and tissue (*in vitro*) and pollutant-associated elevations in systemic IL-6 in subjects with preexisting chronic inflammatory conditions (Arsalane et al. 1995; Carter et al. 1997; Devlin et al. 1991; Dubowsky et al. 2006; Nordenhäll et al. 2000; Quay et al. 1998; Rückerl et al. 2006), although this association has not been demonstrated in young, healthy subjects.

Similar to IL-6, elevated levels of fibrinogen have been shown to be an independent risk factor for cardiovascular disease (Fibrinogen Studies Collaboration 2005; Kannel et al. 1987) and have also been hypothesized to be associated with air pollution (Chuang et al. 2007; Pekkanen et al. 2000; Rückerl et al. 2007; Seaton et al. 1999; Steinvil et al. 2008). This hypothesis is supported by literature considering air pollution and disturbances in hemostasis in general (Baccarelli et al. 2007, 2008; Nemmar et al. 2006), although studies considering the specific association between air pollutants and fibrinogen report conflicting results (Chuang et al. 2007; Pekkanen et al. 2000; Rückerl et al. 2007; Seaton et al. 1999; Steinvil et al. 2008).

Human exposure studies aim to determine the biological effects of air pollutants by using controlled levels of pollutants coupled with an extensive exposure characterization and detailed measurements of physiologic outcomes. For example, controlled human exposure studies using fine concentrated ambient particles (CAPs) have demonstrated changes in biomarkers associated with systemic inflammation, including IL-6 and fibrinogen (Gong et al. 2003, 2004, 2008; Petrovic et al. 2000).

In this study we investigated the association between ambient air pollution and inflammatory markers using baseline data from chamber study exposures. We considered the effects of ozone, nitrogen dioxide, sulfur dioxide, and particulate matter ≤ 2.5 μm in aerodynamic diameter (PM_2.5_) on IL-6 and fibrinogen, using single-pollutant models to analyze repeated-measures data in a group of healthy subjects. We investigated the temporal association between exposure to the pollutants and changes in IL-6 and fibrinogen using moving averages up to 7 days.

In addition to addressing the question of what effect, if any, ambient air pollution exposure has on the baseline levels of IL-6 and fibrinogen, the findings of this study may have additional relevance to the interpretation of human chamber exposure studies: If ambient pollution exposure significantly effects subjects’ baseline inflammatory markers, then pollutant exposure before controlled human exposure studies including CAPs may modify subjects’ inflammatory responses. Before investigating possible effect modification by ambient pollution exposure on human chamber exposure study outcomes, it is first necessary to address what effect, if any, ambient pollution exposure has on subjects’ baseline inflammatory markers, and this was the primary goal of this study.

## Materials and Methods

### Study population

The study population consisted of 45 adults 18–40 years of age who participated in one of two studies conducted in Toronto, Ontario, Canada, between 14 July 1999 and 25 February 2003 (study A) and between 8 January 2004 and 27 March 2006 (study B) and who had IL-6 and fibrinogen measurements at baseline before human chamber exposure to air pollution. Exclusion criteria common to both studies were based on clinical situations or drugs that had the potential to alter systemic inflammation and vascular reactivity. Subjects were excluded if they had a history of coronary artery disease, previous myocardial infarction, peripheral vascular disease, angina, heart failure, or atherosclerosis or used any medications associated with cardiovascular disorders. For the asthmatic participants (*n* = 10), additional exclusion criteria were a forced vital capacity or forced expiratory volume in 1 sec of < 75% of the predicted normal value, or use of oral medications or inhaled corticosteroids for asthma. Bronchodilators were withheld from midnight before each visit. Subjects were also requested to refrain from taking any vitamins or over-the-counter preparations in the 48 hr before each visit.

### Study design

We conducted a retrospective analysis using repeated-measures data collected as part of two controlled human exposure investigations. The data used in this study consisted of the baseline measurements taken before subjects entered the exposure chamber. Height and weight measurements were taken during the initial visit. Blood draws were conducted at 1000 hours on the day of each visit for all participants. All visits were in random order and separated by at least 2 weeks.

This study was approved by the St. Michael’s Hospital Research Ethics Board. The studies from which data were derived complied with relevant research ethics guidelines as well as the Ontario Personal Health Information Act (Personal Health Information Act 2004). Human participants gave written informed consent before enrollment in the study.

### Blood analyses

Venous blood was collected in EDTA tubes for IL-6 and sodium citrate tubes for fibrinogen. Samples were centrifuged, and the resulting plasma samples were stored at –70°C until analysis. Depending on the study for which the sample was drawn, samples were analyzed for IL-6 using either commercial high- sensitivity enzyme-linked immunosorbent assay (ELISA; Amersham Pharmacia, Little Chalfont, Buckinghamshire, UK) or the multiplex Luminex bead assay method (Luminex, Austin, TX, USA). Fibrinogen concentration was determined quantitatively based on the Clauss clotting method (MLA 1600; Medical Laboratory Automation, Pleasantville, NY, USA).

### Exposure measurements

Hourly ambient pollutant data (O_3_, NO_2_, SO_2_, and PM_2.5_) were obtained from an Ontario Ministry of the Environment fixed-site air-monitoring station located in downtown Toronto. Hourly temperature and humidity data, collected at a fixed-site air-monitoring station at Pearson International Airport in Toronto, were provided by Environment Canada.

### Statistical analysis

We conducted univariate explorations for all variables using histograms, linear graphing techniques, and summary statistics. We calculated Spearman correlation coefficients for the pollutant and meteorologic data to investigate the relationships among these variables over the study period. The associations between the inflammatory markers, IL-6 and fibrinogen, and the pollutants, O_3_, NO_2_, SO_2_, and PM_2.5_, were analyzed using linear mixed-effects models with random patient intercepts to account for repeated measures. Date, day of the week, season, and 24-hr moving averages for relative humidity and temperature were included in the models as time-varying confounders based on goodness-of-fit as determined using Akaike’s information criterion. Age, sex, body mass index (BMI), and asthmatic status were included as time-invariant participant characteristics associated with the mean levels of the inflammatory markers to permit the assumption of a normally distributed random patient intercept. Age and date were included in the models as linear variables. We considered data for each of the pollutants at the time of blood draw (baseline) along with the 2-, 4-, 6-, and 12-hr and 1- to 7-day moving averages of the pollutants. We assessed effect modification using an interaction term in the models while also including the main effects of season and pollution. The interaction term consisted of a four-level categorical term for season multiplied by the pollutant being analyzed.

IL-6 data from each study were not directly comparable because study A analyzed samples using ELISA plates whereas study B used the Luminex bead assay system. To allow compilation of the IL-6 data into a single data set, the data were transformed to standard scores (*Z*-scores). As a result, effect estimates for IL-6 are presented as the linear change in the SD of IL-6 per each pollutant’s interquartile range (IQR). Fibrinogen levels were measured using the same techniques in both studies, so effect estimates for fibrinogen are presented as the linear increase in fibrinogen (grams per liter) per each pollutant’s IQR.

Data were analyzed using the statistical package SAS version 9.1 (SAS Institute Inc., Cary, NC, USA).

## Results

### Study population

The study population consisted of 45 participants (22 males, 23 females) with a mean of 3.9 (range, 1–6) repeated blood samples, mean age of 26.6 years (range, 19–48 years), and mean BMI of 22.7 (range, 17.8–29.9). Ten of the 45 subjects had a history of well-controlled asthma.

### Inflammatory markers

Table 1 displays summary data on the inflammatory markers by study and participant characteristics. A total of 163 samples of IL-6 and 160 samples of fibrinogen were available for analysis. IL-6 and fibrinogen were moderately correlated (Spearman correlation coefficient = 0.21, *p* = 0.01).

### Pollutant data

The mean (range) meteorologic and pollutant data were as follows: temperature, 9.2°C (–24.6 to 37.2°C); relative humidity, 69.6% (15–100%); O_3_, 21.9 ppb (0–136 ppb); NO_2_, 23.8 ppb (0–106 ppb); SO_2_, 3.5 ppb (0–70 ppb); carbon monoxide, 0.7 ppm (0–4 ppm); and PM_2.5_, 8.5 μg/m^3^ (0–140 μg/m^3^). These levels are comparable to those reported for major cities in Europe and the United States (Dubowsky et al. 2006; Rückerl et al. 2007). Table 2 displays the meteorologic and pollutant data stratified by season.

Table 3 displays correlations for the pollutants and meteorologic parameters. We observed significant correlations among NO_2_, SO_2_, CO, and PM_2.5_. NO_2_, SO_2_, and CO were negatively correlated with O_3_.

### Regression results

**Figure 1:**
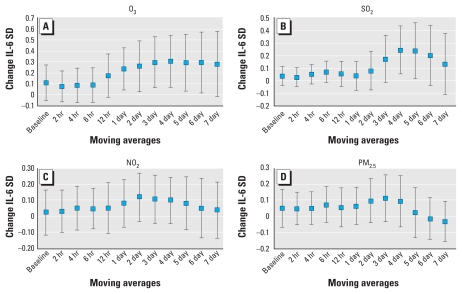
Associations between IL-6 and (*A*) O_3_, (*B*) SO_2_, (*C*) NO_2_, and (*D*) PM_2.5_, per IQR. All models were adjusted for age, sex, BMI, asthma, day of the week, season, temperature (24-hr moving average), and relative humidity (24-hr moving average). Data are mean changes in IL-6 SDs with 95% CIs.

**Figure 2:**
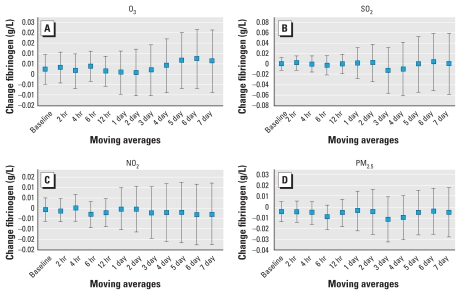
Associations between fibrinogen (g/L) and O_3_ (*A*), SO_2_ (*B*), and NO_2_ (*C*) per 1 ppb increase, and PM_2.5_ (*D*) per 1 μg/m^3^ increase. All models were adjusted for age, sex, BMI, asthma, day of the week, season, temperature (24-hr moving average), and relative humidity (24-hr moving average). Data are mean changes in fibrinogen with 95% CIs.

IL-6 was positively correlated with each of the pollutants investigated and reached statistical significance at the 95% level with O_3_ and SO_2_ (Figure 1). Associations increased with longer moving averages, with statistical significance being reached with the 1- to 6-day moving average for O_3_ and the 4- and 5-day moving average for SO_2_. We observed similar trends for NO_2_ and PM_2.5_. We observed the strongest association between IL-6 and O_3_ using the 4-day moving average: a 0.31 SD increase in IL-6 per O_3_ IQR [95% confidence interval (CI), 0.08–0.54]. The 4-day moving average for SO_2_ showed a 0.25 SD increase in IL-6 per SO_2_ IQR (95% CI, 0.06–0.43). The positive association between IL-6 and each pollutant declined with moving averages longer than 6 days. Fibrinogen was not significantly correlated with any of the pollutants investigated, and we found no trend for increasing moving averages (Figure 2).

### Effect modification by season

**Figure 3:**
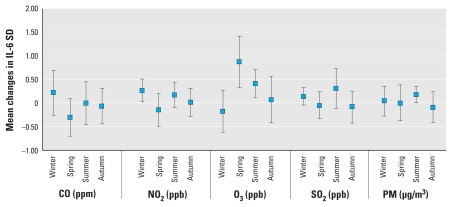
Effect modification of associations between IL-6 and CO, NO_2_, O_3_, SO_2_, and PM_2.5_ by season using 2-day moving averages. All models were adjusted for age, sex, BMI, asthma, day of the week, temperature (24-hr moving average), and relative humidity (24-hr moving average). Data are mean changes in IL-6 SD with 95% CIs for an IQR increase in each pollutant.

Pollution associations with level of IL-6 varied by season, with higher effects of O_3_ on IL-6 in the spring and summer and higher effects of PM_2.5_ in the summer. NO_2_ associations with IL-6 were elevated both in summer (marginally) and in winter. The significance of the effect modification increased with moving averages up to 3 days, with the strongest effects occurring using 2-day moving averages (Figure 3). We observed no effect modification by season for fibrinogen and the investigated pollutants.

## Discussion

In measurements taken before controlled chamber exposure to pollution, study participants had elevated systemic levels of IL-6 in response to elevations in the previous 4-day cumulative averages of ambient O_3_ and SO_2_ levels. IL-6 responses tended to be higher in the spring and summer for O_3_ and in the summer for PM_2.5_. In Toronto these are seasons when open windows may allow more penetration of O_3_ (Ren et al. 2006; Stafoggia et al. 2008) and when mixtures of O_3_ and particulate pollution may be more prominent.

The observed correlations for the pollutants and meteorologic parameters in this study (Table 3) were consistent with the primary sources of the pollutants in the study region and established atmospheric chemical processes. The negative correlation between O_3_ and NO_2_ is in keeping with the process of nitric oxide scavenging O_3_ in the atmosphere and the photodissociation of NO_2_ to nitric oxide and O_3_ (Beaney and Gough 2002). The correlation among CO, SO_2_, and NO_2_ suggests traffic as the common source. This premise is also supported by the fact that the hour-of-day effect for each pollutant peaked in the mornings and, to a lesser extent, the afternoons and the day-of-week effect showed a drop on weekends.

The literature documents pollutant-associated elevations in pulmonary IL-6 in healthy subjects (*in vivo*) and tissue (*in vitro*) (Arsalane et al. 1995; Carter et al. 1997; Devlin et al. 1991; Nordenhäll et al. 2000; Quay et al. 1998; Rückerl et al. 2007). Elevations in systemic IL-6 with elevated PM pollution have also been found in subjects with preexisting chronic inflammatory conditions (Dubowsky et al. 2006). Our study adds to the conclusion that pollution may increase systemic inflammation even in young healthy subjects. In young people, beyond the acute subclinical effects, recurrent low-grade acute inflammatory responses to pollution may ultimately have implications for the evolution of atherosclerosis and other processes influenced by inflammation (Künzli et al. 2005).

Fibrinogen was not significantly associated with any of the pollutants considered in this investigation. It may be that ambient levels of PM_2.5_ in this study were too low to induce a significant effect. This hypothesis is supported by the literature, which has reported no significant association between ambient pollution and fibrinogen at low levels of exposure (Pope et al. 2004; Rückerl et al. 2006) but significant associations with high exposures such as during high air pollution episodes or controlled human exposure studies (Ghio et al. 2000).

In our study of young healthy subjects, we found that cumulative exposure to pollution over longer periods of time (3–6 days) was associated with the strongest associations with elevated IL-6. In elderly subjects with diabetes or obesity, longer cumulative averages also resulted in the greatest effects on inflammation in a study of PM_2.5_ effects on CRP, IL-6, and white blood cells (Dubowsky et al. 2006). Rückerl et al. (2007) found an increase in IL-6 associated with particle number concentration, with a shorter lag of 12–17 hr. It may be that because all of our blood draws took place at 1000 hours, the 2- to 12-hr moving averages represent times when most subjects were in their homes; this may have attenuated the more immediate effects of outdoor pollution levels. Alternatively, at these levels of pollution, a longer cumulative exposure may be needed for this inflammatory response.

A limitation of this study was the small sample size, which limited power to test for interactions. Additional limitations include the absence of indoor home monitoring and the use of fixed-site ambient pollution monitoring, which may result in exposure misclassification, particularly for ambient pollutants that have strong local sources (Briggs et al. 1997). Each of these limitations would be expected to bias the results toward the null. Although we found that the association of many of the pollutants with elevated IL-6 was greater in the summer, correlation among the pollutants and limited numbers of observations made it difficult to evaluate which of the pollutants, or which mixture of pollutants, was leading to the increased inflammatory response. We could not confirm the associations of ambient pollution with inflammation by using additional complementary end points (e.g., CRP, tumor necrosis factor-α). We are able to describe relative but not absolute changes of IL-6 levels in response to pollution because we transformed the data to *Z*-scores in order to combine data from two separate studies.

The potential implications of the findings of this study extend beyond demonstrating that ambient pollutant exposure has a significant effect on baseline systemic levels of IL-6. Knowledge regarding the effect of prior ambient pollution exposure in baseline evaluation of systemic inflammation in human chamber exposure studies is important for interpretation of the results of such studies. Ning et al. (2004), using *in vitro* studies, demonstrated that “priming” of lung epithelial cells with inflammatory mediators before exposure to fine CAPs resulted in a greater inflammatory response. By analogy, priming of baseline inflammatory status by prior ambient air pollution exposure could modify the response of human subjects to controlled pollutant exposure in chamber studies. Such effect modification could lead to nondifferential misclassification bias (trials are conducted in random order), resulting in an underestimation of the true effect that pollutant exposure has on the induction of inflammation in humans.

Alternatively, ambient pollution could potentially prime and amplify the response to exposure in the chamber. A third possibility is that adaptation through ambient exposures to pollution could dampen certain immunologic or physiologic responses to acute chamber exposures (Bell et al. 1977). Finally, if prior exposure to ambient pollution were to equally affect pre- and postexposure measurements, then there would be much less concern about taking prior exposures to ambient pollutants into effect when doing controlled exposure studies. Investigation of the effects of cumulative ambient exposures to pollutants on responses to controlled human chamber exposure to pollutants will help in the interpretation of these studies.

## Conclusion

Our results support previous findings of an association between ambient pollution and IL-6. In our analysis, exposure to ambient levels of O_3_ and SO_2_ was positively and significantly associated with a systemic inflammatory response as measured by systemic levels of IL-6. The association between IL-6 and O_3_ and SO_2_ demonstrated a cumulative lag effect with the strongest effects observed using 3- to 5-day moving averages. Pollution effects varied by season. Fibrinogen levels were not correlated with any of the investigated pollutants. Having demonstrated an effect of ambient pollutant exposure on baseline systemic levels of IL-6, future research should focus on whether ambient pollution exposure modifies the effect of inflammatory responses to controlled pollution exposures human chamber studies.

## Figures and Tables

**Table 1:** Inflammatory markers by study and participant characteristics.

Study/characteristic	No. of subjects	Mean fibrinogen (range) (g/L)	Mean IL-6 (range) (pg/mL)
Study A			
All participants	23	2.30 (0.97–3.80)	0.64 (0–2.67)
Female	12	2.47 (1.50–3.80)	0.50 (0.02–2.40)
Male	11	2.09 (0.97–2.93)	0.82 (0.00–2.67)
Nonasthmatic	13	2.29 (0.97–3.53)	0.75 (0.00–2.67)
Asthmatic	10	2.32 (1.38–3.80)	0.50 (0.05–1.84)

Study B			
All participants	22	2.54 (1.38–4.55)	39.06 (3.97–146.20)
Female	11	2.83 (1.87–4.36)	48.93 (4.52–138.30)
Male	11	2.35 (1.38–4.55)	33.38 (3.97–146.20)

Study A analyzed IL-6 samples using ELISA plates, whereas study B used the Luminex bead assay system. IL-6 data were transformed to standard scores (*Z*-scores) before compilation into single data set.

**Table 2:** Pollutant and meteorologic data by season, 14 July 1999 to 27 March 2006 (mean ± SD).

Pollutant	Spring	Summer	Autumn	Winter	Annual
O_3_ (ppb)	26.42 ± 13.55	30.80 ± 18.76	16.89 ± 13.40	13.78 ± 9.27	21.94 ± 15.78
NO_2_ (ppb)	24.98 ± 13.36	20.83 ± 11.54	22.61 ± 11.19	26.78 ± 10.70	23.79 ± 11.95
SO_2_ (ppb)	3.09 ± 11.40	2.95 ± 13.62	3.61 ± 3.60	4.60 ± 4.67	3.57 ± 9.31
PM_2.5_ (μg/m^3^)	7.52 ± 7.29	12.34 ± 11.26	7.70 ± 8.05	6.12 ± 4.98	8.46 ± 8.57
Temperature (°C)	7.47 ± 7.86	21.11 ± 4.88	10.93 ± 7.22	–2.85 ± 6.17	9.22 ± 10.82
Humidity (%)	64.20 ± 17.83	66.69 ± 16.39	73.31 ± 15.13	74.25 ± 12.41	69.58 ± 16.16

**Table 3:** Meteorologic and pollutant data (daily averages): Spearman rank correlation coefficients for the study period 14 July 1999 to 27 March 2006.

	CO	NO_2_	O_3_	SO_2_	PM_2.5_	Humidity	Temperature
CO	1.00	0.49	−0.24	0.43	0.25	−0.06	−0.10
NO_2_		1.00	−0.53	0.44	0.41	−0.11	−0.19
O_3_			1.00	−0.19	0.03**	−0.23	0.32
SO_2_				1.00	0.45	−0.10	−0.09
PM_2.5_					1.00	0.10	0.42
Humidity						1.00	0.05
Temperature							1.00

All correlations *p* < 0.01 unless otherwise specified.

** *p* = 0.148.
